# Transactions of the Medical Society of Pennsylvania

**Published:** 1889-02

**Authors:** 


TRRANACTIONS OF THE MEDICAL SOCIETY OF PENNSYLVANIA. Thirty-ninth Annual Session, held in Pennsylvania, June, 1888.

This volume is bound in cloth and contains 355 pages in clear type. The President is James B. Murdoch, M. D., of Allegheny county, and the Secretary is W. B. Atkinson, M. D., of Philadelphia. About thirty papers of more than an average in volume are to be found in this book. Medical legislation comes in for a good share of discussion.—B.

